# Fungal association and utilization of phosphate by plants: success, limitations, and future prospects

**DOI:** 10.3389/fmicb.2015.00984

**Published:** 2015-10-16

**Authors:** Atul K. Johri, Ralf Oelmüller, Meenakshi Dua, Vikas Yadav, Manoj Kumar, Narendra Tuteja, Ajit Varma, Paola Bonfante, Bengt L. Persson, Robert M. Stroud

**Affiliations:** ^1^School of Life Sciences, Jawaharlal Nehru UniversityNew Delhi, India; ^2^Institute of Plant Physiology, Friedrich-Schiller-University JenaJena, Germany; ^3^School of Environmental Sciences, Jawaharlal Nehru UniversityNew Delhi, India; ^4^Plant Molecular Biology Group, International Center for Genetic Engineering and BiotechnologyNew Delhi, India; ^5^Institute of Microbial Technology, Amity UniversityNoida, India; ^6^Department of Biology, University of TorinoTorino, Italy; ^7^Centre for Biomaterials Chemistry, Department of Chemistry and Biomedical Sciences, Linnaeus UniversityKalmar, Sweden; ^8^Department of Biophysics and Biochemistry, University of California at San Francisco, San FranciscoCA, USA

**Keywords:** phosphates, transporters, rhizosphere, phosphate transport proteins, phsophate uptake

## Abstract

Phosphorus (P) is a major macronutrient for plant health and development. The available form of P is generally low in the rhizosphere even in fertile soils. A major proportion of applied phosphate (Pi) fertilizers in the soil become fixed into insoluble, unavailable forms, which restricts crop production throughout the world. Roots possess two distinct modes of P uptake from the soil, direct and indirect uptake. The direct uptake of P is facilitated by the plant’s own Pi transporters while indirect uptake occurs via mycorrhizal symbiosis, where the host plant obtains P primarily from the fungal partner, while the fungus benefits from plant-derived reduced carbon. So far, only one Pi transporter has been characterized from the mycorrhizal fungus *Glomus versiforme*. As arbuscular mycorrhizal fungi cannot be cultured axenically, their Pi transporter network is difficult to exploite for large scale sustainable agriculture. Alternatively, the root-colonizing endophytic fungus *Piriformospora indica* can grow axenically and provides strong growth-promoting activity during its symbiosis with a broad spectrum of plants. *P. indica* contains a high affinity Pi transporter (PiPT) involved in improving Pi nutrition levels in the host plant under P limiting conditions. As *P. indica* can be manipulated genetically, it opens new vistas to be used in P deficient fields.

## Introduction

Nutrients play essential roles in cell metabolism since limitations restrict cell growth, development and productivity. One of the major nutrients is Pi but in most soils Pi is not available as it is immobilized by adsorption, P mineralization and fixation into organic molecules (Marschner, 1995). An available form of P for plants is soluble orthophosphate (Pi), but its concentration in the soil solution hardly reaches 10 μM, and Pi is often present in sub-micromolar levels at the root-soil interface. The area around the root surface, where active Pi acquisition takes place, is called the Pi depletion zone (Lewis and Quirk, 1976). This zone is persistent due to slow diffusion of Pi from other unexplored areas. When plants are unable to acquire sufficient amounts of Pi, they perform morphological and physiological changes in the roots to increase soil exploration and the total absorptive surface area. These changes include extensive root branching, increase in length of root hairs (Raghothama, 1999), and the activation of an advanced bio-molecular system, which results in enhanced P absorption. Furthermore, enhanced acid and acid phosphatase (rAPase) secretion and expression of a new kind of Pi transporter in the root cell that is highly efficient in Pi acquisition under P-limiting conditions are typical features of roots under Pi limitation.

P deficiency shifts the priority of energy expenditure from growth, development and reproduction to P acquisition, a situation that adversely affects the plant productivity. To overcome or minimize the effect of P shortage, plants associate with AMF as obligate biotrophs (Parniske, 2008; Bonfante and Genre, 2010), but also other beneficial root-colonizing fungi such as *Piriformospora indica*.

## P Fertility in Soil and Accessibility for the Plants

P is one of the 17 essential elements required for plant growth and development (Bieleski, 1973; Raghothama, 1999). The P concentration in plants ranges from 0.05 to 0.5% of the dry weight. The Pi concentration gradient from the soil to plant cells increases more than 2,000-fold, with an average physiological concentration of 10 μM in the soil (Schachtman et al., 1998). P is a highly reactive element and thus does not exist in the elemental form in nature. Although P is quite abundant it is mainly present as Pi in insoluble complexes with cations, particularly aluminum and iron under acidic conditions. Only 10–15% of the total P is present as soluble Pi (Hinsinger, 2001). Because of this, the crop yield on 30–40% of the world’s arable land is limited by the P availability (Runge-Metzger, 1995; Von Uexkull and Mutert, 1995). The continuous depletion of terrestrial P in the soil is counteracted by the use of fertilizers (Raghothama, 1999; Gilbert, 2009), which contain P mainly as Pi. However, up to 80% of supplied Pi is again fixed in insoluble complexes leading farmers to use up to four times more fertilizer than required for optimal crop production (Barrow, 1980; Goldstein, 1992; Holford, 1997). Furthermore, in the soil, P exists as also organic Pi and phytates (**Box 1**), and the solubility of these unaccessible P from is largely dependent on the pH of the rhizosphere, which changes locally and depends on the microbial community in the root environment (Haynes, 1982; Robinson, 1994; Marschner, 1995; Rengel and Marschner, 2005).

BOX 1 **Availability of total Pi in the soil. Out of three forms present in the soil, plant utilizes only inorganic Pi.**

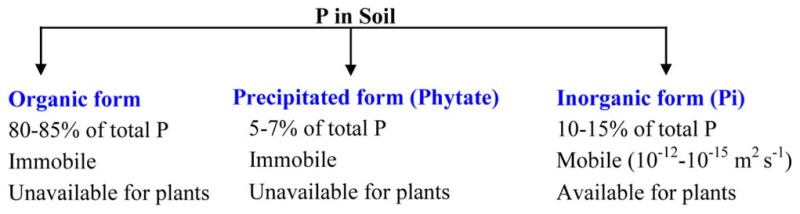

P is taken up by the roots as either monovalent H_2_PO_4_^-^ or to a lesser extent as secondary HPO_4_^2-^ (Ullrich-Eberius et al., 1984; Furihata et al., 1992). H_2_PO_4_^-^ is dominant in acid soils and taken up about 10 times more efficiently than HPO_4_^2-^. At a soil pH of 7 approximately equal amounts of the two Pi forms are present, and the secondary ortho-Pi ion becomes the dominant form above pH 7. In extremely acidic or alkaline soils the solubility of Pi is decreased, with the dominant forms being H_3_PO_4_ or PO_4_^3-^, respectively, (Brady, 1990). Inositol Pi in soil is represented by its hexa-Pi esters, generally called phytates, which constitute up to 60% of soil organic P and often form salts with different ions (Stevensons, 1994; Jennings, 1995). Because phytates are less soluble, they cannot be utilized by plants (Pearson et al., 1941; Jennings, 1995).

## P Acquisition by Plants: Secret Hidden Below the Ground

A balanced Pi metabolism requires Pi acquisition, translocation from roots to shoots, as well as distribution and remobilization within the plant (Lauer et al., 1989; Liu et al., 2010). Generally, plants maintain a threshold cytoplasmic Pi concentration regardless of the variation in external concentration, which is necessary for safeguarding the primary and secondary metabolism. For short term P security, plants store P as poly Pi in the cytoplasm or vacuoles (Bieleski, 1973; Mimura et al., 1990, 1996; Tu et al., 1990; Sakano et al., 1992).

Pi is supplied to the roots by diffusion rather than mass flow, and the rate of diffusion of Pi is slow in soil. To meet the plant’s demand for soluble P from the rhizosphere, the soil solution should be replaced 20 to 50 times per day (Marschner, 1995). The ion absorption occurs primarily at the young root tip, before uptake into the epidermal cells of the root hairs and apoplastic absorption through the outer layers of the cortical cells. The apoplastic uptake/capture of Pi from the soil is the critical step before the transport into the cells by an energy-driven mechanism (Mikkelsen, 2005). The interlaced fibers of the cell wall form an open latticework in roots that serves as a sieve for the soil solution. The apoplast allows the soil solution to move into the tissue until it reaches the Casparian strips. Because the cell wall fibers have a net negative charge, they repel anions such as Pi and nitrate in solution and confine their transport to larger pores within the apoplastic space. Some root secretions, such as mucilage (an organic compound secreted by the root cells) also bear an overall negative charge that can work as an additional anion repellant from the root (Mikkelsen, 2005). The symplastic route of ion transport is rather complex and connected to all living cells through plasmodesmata. The epidermis and the outer cortical layer pass the ion to the inner adjacent cortical cells.

**FIGURE 1:**
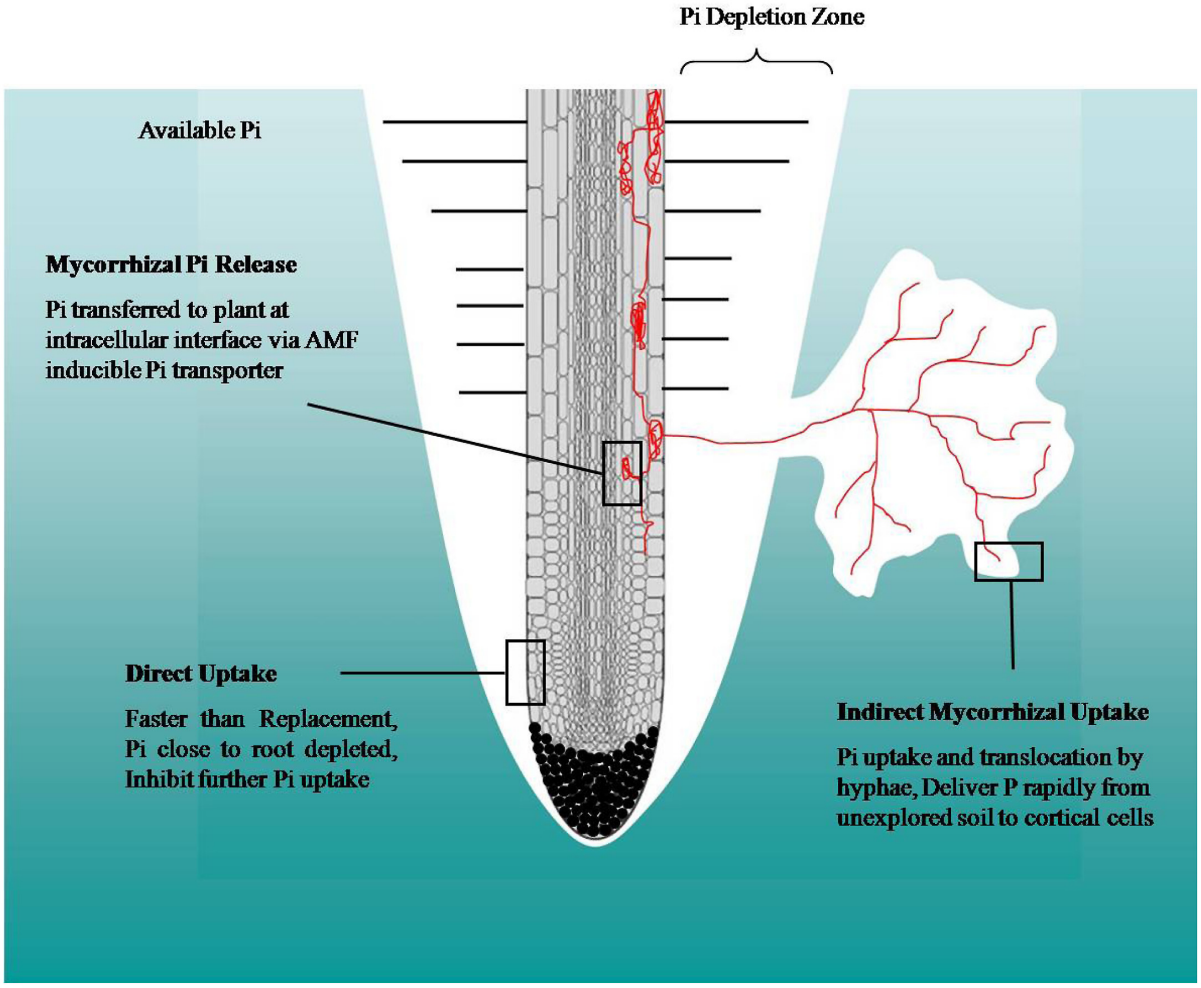
**Uptake of phosphorus by plants from soil.** Two modes of P uptake are present in plants. **(A)** Direct uptake – between soil and plant root and **(B)** indirect uptake – through mycorrhizal symbiosis, where AM fungus extra-radical mycelium acquire Pi beyond rhizosphere and Pi transported to intra-radical mycelium as poly-phosphate (Rosewarne et al., 1999).

AMF increase the absorptive area (Bolan, 1991; Smith and Read, 1997), because the fine and thinner structure of the fungal hyphae have better access to soil pores and can explore larger soil volumes, which results in more efficient mining for Pi sources (Rosewarne et al., 1999; Drew et al., 2003; Smith and Read, 2008; Schnepf et al., 2011; **Figure 1**).

Root hairs are the primary site for the Pi acquisition and in response to Pi scarcity both the density and length of root hairs increase to explore a larger volume of soil. P-deficient plants are characterized by increases in root/shoot ratio, root branching, root elongation, and root top soil exploration. The root hairs are commonly longer, while primary root growth is reduced (Lynch, 1995; Ma et al., 2001a,b; Lopez-Bucio et al., 2003; Lynch and Brown, 2008; Vance, 2010). The development of dense lateral root clusters with large numbers of root hairs has been observed in white lupine (*Lupinus albus*; Wang et al., 2010).

Besides acidification of the rhizosphere, exudation of citrate, malate, or oxalate greatly enhances mobilization of Pi by chelation or ligand exchange (such as Ca, Fe, or Al). Root-induced acidification can decrease the rhizosphere pH by 2–3 units relative to the bulk soil (Marschner, 1995). Secretion of phosphatases or phytases mobilize organic Pi through hydrolysis (Vance et al., 2003; Hinsinger et al., 2005; Popova et al., 2010; Zhang et al., 2010). A major problem in P acquisition is the uptake of Pi against a concentration gradient, and this requires specific high-affinity Pi transporters and energy. The symport requires a proton gradient and the electrochemical gradient across the plasma membrane is generated by P-type H^+^ ATPases at the expense of ATP. The changes of the root architecture under P limitation have a strong influence on the carbohydrate metabolism and their distribution between roots and shoots, and these changes often involve plant hormone (Nacry et al., 2005; Rohmeld and Newmann, 2006; Wang et al., 2010) and sugar signaling (Karthikeyan et al., 2007; Vance, 2010). The formation of a highly branched root system in response to Pi starvation is a consequence of the canalization of carbon and energy resources to the root surface (Stitt and Rudige-Scheible, 1998). Root exudation of organic acids and enzymes ultimately causes a loss of carbon that results in loss of crop yield under Pi limitation.

## Molecular Mechanism of P Uptake

**FIGURE 2:**
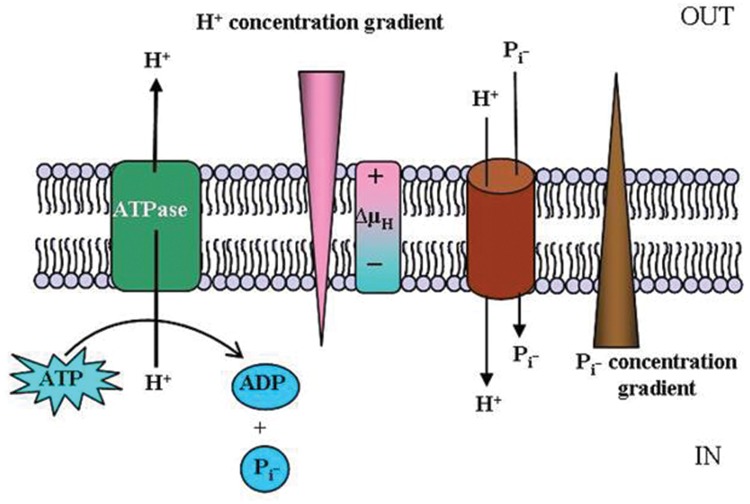
**Pi uptake mechanism across the plasma membrane.** A membrane-integral proton ATPase uni-directionally extrudes protons (H^+^) at the expense of ATP (primary transport). The generated proton concentration gradient and membrane potential constitute a proton electrochemical potential (Δμ_H_) across the membrane. Proton movement along concentration and electrical gradients facilitates Pi (Pi^-^) allocation through Pi transporters against a steep concentration gradient (secondary transport; Karandashov and Bucher, 2005).

The existence of high- and low-affinity Pi transport systems have been reported in plants, bacteria, yeast, AMF, endophytic fungi and animals (Burns and Beever, 1977; Thomson et al., 1990; Olah et al., 1994; Zvyagilskaya et al., 2001; Yadav et al., 2010; Pedersen et al., 2013). To overcome the steep concentration gradient (Bieleski and Ferguson, 1983) and negative membrane potential, the cells require an energized transport of Pi across the plasma membrane (Bieleski and Ferguson, 1983). Pi transport transiently depolarizes the plasma membrane, which indicates that Pi does not simply enter the cell as H_2_PO4^-^ or HPO_4_^2-^, both of which would lead to a membrane hyperpolarization if Pi is transported alone (Ullrich-Eberius et al., 1984). An energy-dependent Pi uptake via H^+^/Pi co-transport has been first demonstrated for *Lemna gibba* which depends on the electrochemical proton gradient and therefore on the activity of an H^+^-extrusion pump such as the P-type H^+^-ATPase (Ullrich-Eberius et al., 1981, 1984; **Figure 2**). The large membrane potential difference with a negative potential on the cytoplasmic site (-150 to -200 mV) provides the driving force for co-transport of Pi and other ions with protons (Ullrich-Eberius et al., 1984; Daram et al., 1998; Sze et al., 1999; Karandashov and Bucher, 2005). Hyphae of the ectomycorrhizal *Hebeloma cylindrosporum* have at least two high-affinity Pi transporters (HcPT1 and HcPT2) that are differentially expressed depending on the P availability and mycorrhizal status (Tatry et al., 2009). Further, functional studies of these transporters heterologously expressed in yeast suggest that P uptake into yeast cells is pH sensitive. Disruption of the pH gradient by uncouplers drastically reduced the Pi uptake, which confirmed that these transporters are proton-coupled and indirect energy-dependent symporters (Tatry et al., 2009). In contrast, at higher pH values (9.5–10) H^+^ cannot influence the Pi uptake. Under these conditions, uptake occurs by several Na^+^-dependent transport systems that are kinetically discrete from the H^+^-dependent transport, specifically activated by Na^+^ ions and thus insensitive to the protonophore CCCP. The H^+^-coupled P transport systems provide most, if not all, of the P uptake at pH values of 4.5 and 6.0. The contribution of the Na^+^/Pi co-transport systems to the total cellular P uptake activity progressively increases with increasing pH and reaches its maximum at pH 9 and higher, i.e., conditions where P accumulation was preferentially, if not exclusively, maintained through the Na^+^/Pi co-transport systems. H^+^/Pi co-transport occurred even at pH 8.0, presumably as a consequence of local pH gradients in the vicinity of the carriers in the plasma membrane. At pH 7.0, both H^+^/Pi and Na^+^/Pi co-transport systems are equally responsible for P uptake. The H^+^- and Na^+^-coupled P transport systems thus possess overlapping but distinct biological roles in the acquisition of P under different growth conditions (Zvyagilskaya et al., 2001).

## P Transporters: Entrance of Pi into Cell

When the external P level drops to micro-molar concentrations, the transcript levels for high affinity transporters in roots increase, preferentially in cells with close contact to the soil solution. The low-affinity transporters are mainly active in vascular tissues and involved in the internal distribution and re-mobilization of P (Smith, 2001).

High-affinity Pi transporters have been identified and characterized in several plant and fungal species, including *Arabidopsis thaliana, Medicago truncatula, Lycopersicon esculentum, Solanum tuberosum, Saccharomyces cerevisiae* and *Neurospora crassa* (Bun-Ya et al., 1991; Olah et al., 1994; Versaw, 1995; Munchhal et al., 1996; Leggewie et al., 1997; Daram et al., 1998; Liu et al., 1998a,b; Rausch et al., 2001). Plant Pi transporters are grouped into three families: the Pht1 family which contains high-affinity transporters, the Pht2 family which contains transporters responsible for Pi translocation, and the Pht3 family for plastid and mitochondrial P transporters. The fungal Pi transporters are an extension of the Pht1 family (Karandashov et al., 2004; Karandashov and Bucher, 2005). Phylogenetically, they are closely related proteins, although the similarity between the plant transporters is higher than between plant and fungal transporters (Munchhal et al., 1996). The transporters of this family are 500–600 amino acids long and contain 12 predicted membrane-spanning hydrophobic regions of 17–25 amino acid residues which are arranged in a helix. The membrane spanning regions are arranged in two groups of six well defined configurations with a large central hydrophilic, charged loop. This topology is shared by fungal, yeast, plant, and animal Pht1 family members as well as several members of other transporter families (Saier and Reizer, 1991; Pao et al., 1998).

**FIGURE 3:**
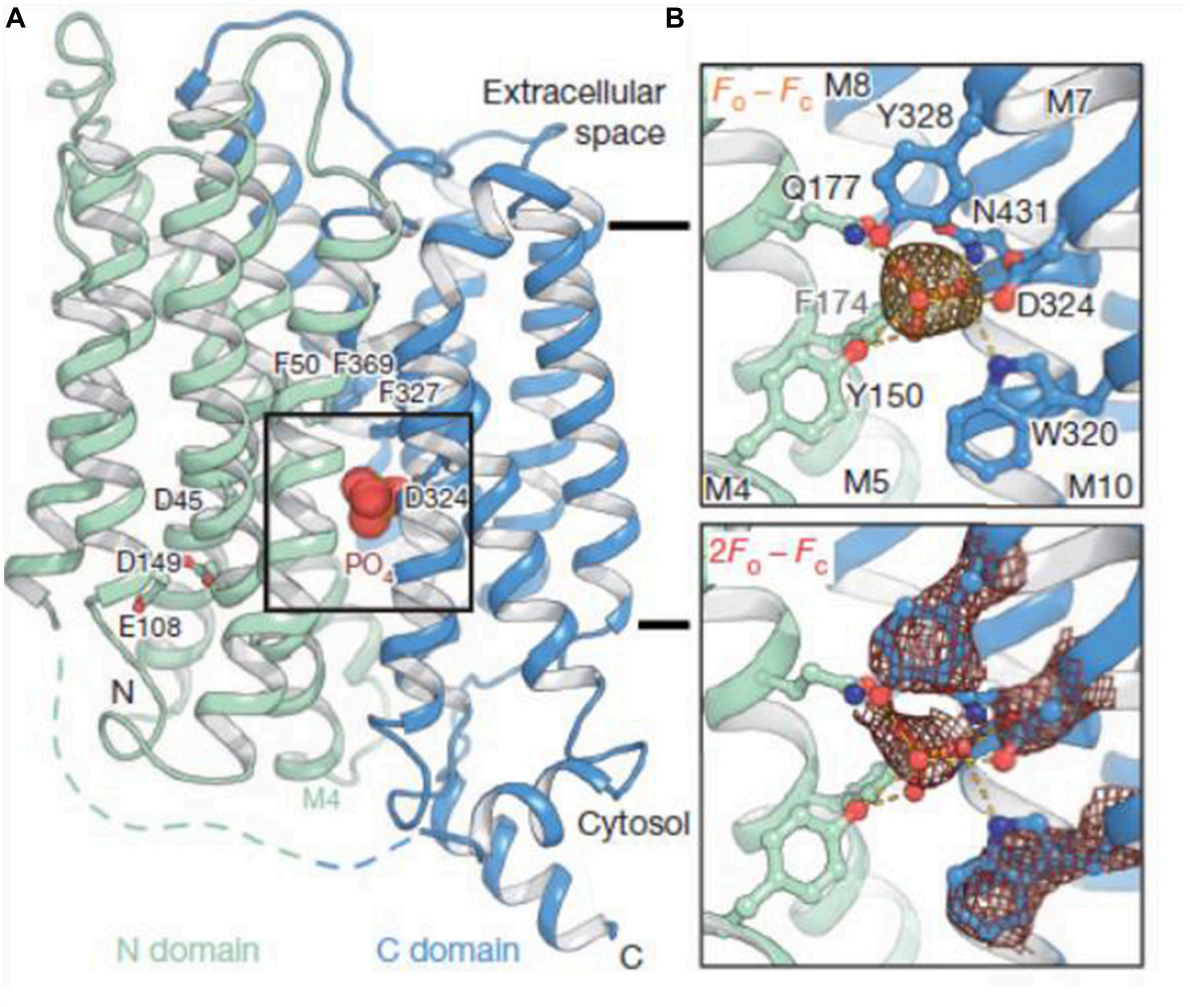
**Structure of the high-affinity phosphate transporter, PiPT.** The structure represents an inward facing occluded state of the phosphate transporter in complex with phosphate. **(A)** Phosphate (shown as spheres) is buried in the membrane at the interface between the N domain (pale green) and C domain (blue). Selected residues are shown as sticks. Black bars depict the approximate location of the membrane. **(B)** The phosphate-binding site with yellow dashes indicating possible hydrogen bonds (2.2–3.8Å distances) to phosphate. Top: the omit 2mFo-DFc density for phosphate is contoured in orange (4s). Bottom: the 2mFo-DFc density for phosphate and selected M7 residues is contoured in red (2s). Reproduced with permission from Macmillan Scientific Publishers Ltd. (London). For more detailed information on PiPT structure please see reference Pedersen et al. (2013).

Recently, Yadav et al. (2010) demonstrated that the high affinity *P. indica* Pi transporter PiPT improves the Pi nutrition levels in the host plant under P limitation. Furthermore, the crystal structure of PiPT confirms that the Major Facilitator Superfamily (MFS)-fold found in bacteria is conserved in eukaryotes. PiPT has 12 transmembrane helices divided into two homologous N- and C-terminal domains (Pedersen et al., 2013; **Figure 3**). Overall, the conformation of PiPT is similar to structures of transporters of the MFS in their occluded state. The Pi is coordinated by Tyr, Gln, Trp, Asp, and Asn side chains. All these residues are fully conserved in the family of Pi:H^+^ symporters. Asp coordinates the Pi with both carboxyl oxygens (**Figure 3**). The structure of PiPT (Pedersen et al., 2013) explains the structural/functional relationships of Pi/H^+^ symport by providing structural confirmation for Pi affinity and specificity and relating the proton motive force to Pi translocation.

While the Na^+^-dependent P transport system is found in animal cells; the H^+^-coupled Pi transport occurs mainly in plants (Escoubet et al., 1989; Werner and Kinne, 2001). A Na^+^-dependent Pi-uptake system has not yet found in vascular plants, but its existence cannot be excluded especially in halophytes, which have developed mechanisms for salt resistance, or in plants living in alkaline soils. Based on transport measurements, it appears that both types of transporter systems are present in fungal species (Versaw and Metzenberg, 1995; Zvyagilskaya et al., 2001; Pedersen et al., 2013). H^+^-driven P transport in yeast and *N. crassa* was reported to exhibit a pH optimum between 4.5 and 6.0, which is in the range of apoplastic pH values in plants, while Na^+^-coupled co-transport had a pH optimum of 8.0 and higher (up to 10; Versaw and Metzenberg, 1995; Zvyagilskaya et al., 2001). This suggests a high flexibility of these organisms for changing environmental conditions.

## Functional Analysis of P Transporters

The yeast *S. cerevisiae* provides a useful model system for heterologous expression and functional analyses of plant and fungal P transporters. The *pho84* yeast mutant, defective in a gene encoding one of the yeast P transporters (Bun-Ya et al., 1991), has been used for complementation studies. Michaelis constant (*K*m) values for plant P transporters derived by heterologous expression studies in this mutant are generally higher than expected making it difficult to obtain reliable kinetic data (Leggewie et al., 1997; Liu et al., 1998b). Recently, a *S. cerevisiae* double mutant, *PAM2*, became available. This mutant has disruptions in the genes encoding both the H^+^-coupled Pho84 and the Na^+^-coupled Pho89 high-affinity Pi transporters (Martinez and Persson, 1998). The Pho84 transporter is involved in sensing extracellular Pi levels and cytosolic signaling due to its dual function as a transceptor (Popova et al., 2010; Samyn et al., 2012). The activity of the Pho84 transceptor mediates signaling via the PHO pathway and is also involved in the activation of the protein kinase A pathway. Mutational analysis of Pho84 has shown that the residues Asp-358 and Lys-492 are critical for the Pi transport function suggesting that they are part of the substrate-binding pocket. Mutants of Asp-358 in the putative H^+^-binding site are still capable of activating protein kinase A targets, despite a severely hampered transport activity (Samyn et al., 2012). Translocation of H^+^ and Pi relies on an asymmetric ‘rocker-switch’ mechanism compatible with the mechanism of other MFS transporters.

Typical *K*m values of high-affinity systems in plants and fungi are in the range of 5–50 μM for Pi. The values for AMF transporters are likely within the same range, although substantial variations have been shown (Burns and Beever, 1977; Mimura, 1999). For instance, the *K*m values of the high-affinity and low-affinity systems for germ tubes of *Gigaspora marginata* were 1.8–3.1 μM and 10.2–11.3 mM, respectively, (Thomson et al., 1990), while that of *Glomus versiforme (GvPT)* for a high-affinity Pi transporter expressed in the external mycelium of the AM fungus *GvPT* was 18 μM (Harrison and van Buuren, 1995). PiPT is expressed in extra-radical hyphae of *P. indica.* When heterologously expressed in yeast PiPT exhibited an apparent *K*m of 25 mM (Yadav et al., 2010). An apparent *K*m of 31 μM was measured for the tomato *LePT1* Pi transporter using the yeast PAM mutants (Daram et al., 1998). While this may still be higher than expected for a high-affinity Pi transporter, it is an order of magnitude lower than the values measured for plant Pi transporters expressed in the *pho84* mutant. Expression of a cDNA encoding a plant Pi transporter in cultured tobacco cells has yielded the most reliable functional analysis. Mitsukawa et al. (1997) proved that the PHT1 transporter from *Arabidopsis* is a high-affinity P transporter with an apparent *K*m of 31 μM. The sequences and expression patterns of some of the P transporters from *Arabidopsis* and barley are almost identical suggesting that they have similar functions (Smith and Read, 1997; Smith et al., 1999). It seems that at least in some diploid plant genomes, there is a redundancy of genes encoding P transporters that are critical for the P uptake and hence plant performance. The high degree of sequence similarities suggests that the genes are the result of recent gene duplications.

## “The Helping Hands” – P Uptake Through Mycorrhizal Association

In AM associations, plants acquire Pi from the extensive network of extra-radical hyphae of fungi that extend beyond root depletion zones to mine new regions of the soil (Harrison and van Buuren, 1995). The indirect uptake of Pi in mycorrhizal plants results in higher Pi uptake rates than in non-mycorrhizal plants under P limited conditions (Haynes, 1982; Kumar et al., 2011). The role of AMF in nutrient acquisition of their host seems to be inversely related to the development of the root system (Newsham et al., 1995; Schweiger et al., 1995). In nature, there is, however, a variation in P uptake in relation to colonization by different AMF, since isolates differ in P transfer efficiency and also in P supply to the plant (Pearson and Jakobsen, 1993; Joner and Jakobsen, 1995). For instance, *G. aggregatum* provides less and *G. intraradices* more Pi to the same host (Kiers et al., 2011). Interestingly, plants also regulate the fungal growth in the root depending upon the external P status, and at high P supply root colonization seems to be reduced (Thomson et al., 1990).

In case of *GvPT, G. intraradices*, and *G. mosseae*, the function of Pi transporters have been studied in heterologous systems but their role in Pi transportation in AMF could not be verified due to the lack of a stable transformation systems (Maldonado-Mendoza et al., 2001; Harrison et al., 2002; Benedetto et al., 2005). Since the difference in electrochemical potential between soil and extra-radical mycelium is large, being negative in the mycelium, and since the P concentration inside the mycelium is high compared with the soil solution, the absorption of P by AMF must be an active process (Smith and Read, 1997). The first fungal P transporter that is involved in the uptake of P from soil has been identified in *GvPT.* The *GvPT* encodes a high-affinity fungal P transporter that is similar in both structure and function to high affinity transporters of plants (Harrison and van Buuren, 1995). Maldonado-Mendoza et al. (2001) have identified a P transporter gene in the extra-radical mycelium of *G. intraradices* named *GiPT*, which is expressed at low P concentration in the growth substrate. Thus, extra-radical hyphae in a mycorrhiza are involved in the absorption of Pi and other nutrients from the soil, its translocation from the surrounding soil to the hyphae and through the mycelium into the fungal structures within the root (Smith and Read, 1997). Other reports have shown that plant P transporters, inducible by P starvation, are down-regulated in mycorrhizal roots, indicating that these transporters are not involved in symbiotic P transfer (Chiou et al., 2001). It is believed that the initial Pi uptake under Pi limitation into the fungus-plant community is almost entirely due to uptake by the extra-radical fungal hyphae (Pearson and Jakobsen, 1993).

Mycorrhizal associations are not always beneficial for Pi nutrition. It can be costly for the plant and the fungal partners may alter their mutualistic responsibility from beneficial to neutral (Kiers and Van der Heijden, 2006; Douglas, 2008). Consequently, AMF colonization can have no or negative effects on the plant’s growth and performance (Johnson et al., 1997). Depression of plant growth can be due to high C-demand of the fungal partners leading to lack of P benefit for the plant. Poor growth can already occur with low fungal biomass. Furthermore, growth depression can also be the result of reduction in P acquisition through the direct P-uptake pathway of the plant that is not compensated for by the fungal P delivery (Smith et al., 2009, 2011). However, in the plant-mycorrhizal mutualism both symbionts are able to detect variations in the resources supplied by their partners, allowing them to adjust their own resource allocation accordingly. Host plants discriminate between cooperative and less cooperative AMF partners. They may supply them with more or less amounts of carbon, depending on the amount of P they receive from the fungal partner. However, this reciprocal reward between the plant and more cooperative partners depends on carbon supply. The P transfer to the plant is proportional to the C supply (Kiers et al., 2011). Reprogramming of root development under Pi limitation shares similarities with processes known from root colonization by AMF (Rubio et al., 2009), or other beneficial root-colonizing fungi. This offers a great potential for cross-talk studies.

## Mechanism of Indirect P Uptake

P uptake into the mycorrhizal plants cells takes place at a specialized interface between AMF and host cells called arbuscules. When a fungal hypha penetrates a cortical cell and differentiates into an arbuscule, the plant cell plasma membrane extends to surround it with the so-called periarbuscular membrane, localizing the arbuscule essentially to the plant apoplast. The extended periarbuscular membrane including the residual plant cell wall is separated from the fungal wall and the underlying membrane of the AMF by a narrow compartment, the interface matrix (Harrison, 1999).

**FIGURE 4:**
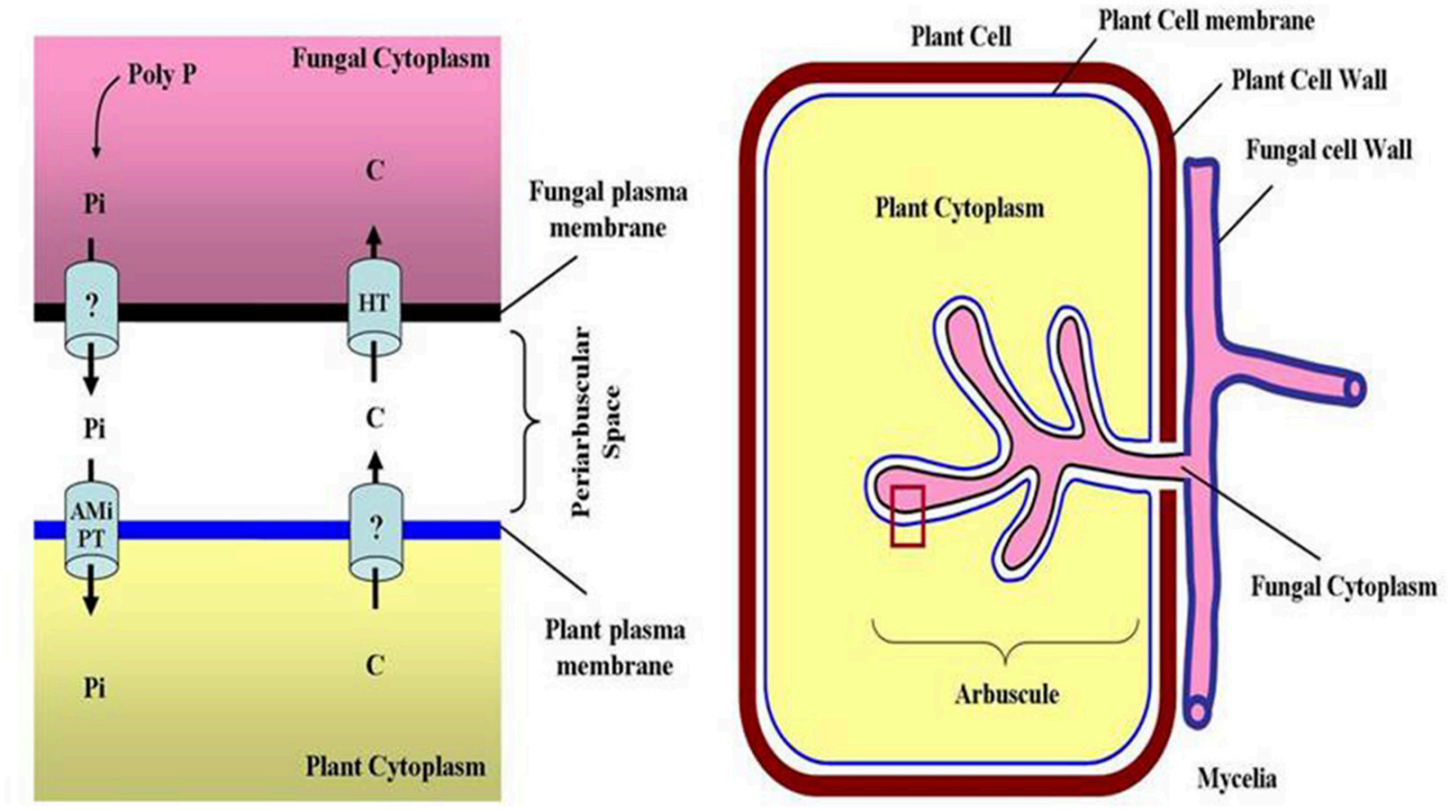
**Translocation of phosphorus **(P)** and carbon **(C)** at fungus – plant interface.** Inorganic P is taken up by specialized transporters located on the fungal membrane in the extra-radical mycelium. Pi (originated in AM fungi from the hydrolysis of polyphosphate) is imported from the symbiotic interface to the plant cells through AM inducible Pi transporters (AMiPT). Hexose transporters (HT) import plant-derived carbon to the fungus, whereas transporter proteins involved in the export of nutrients from plant have not been identified yet.

The indirect pathway of Pi acquisition through AM associations involves (a) uptake of P from the soil across the membrane of the fungal hyphae, (b) movement of P along the hyphae to the arbuscules, (c) unloading the P from the fungal arbuscules at the arbuscule-cortical cell interface, and (d) uptake of that P by the plant cortical cells. The suggested mechanisms of P translocation within the AM fungal hyphae from the place of uptake to the fungus–root interfaces involve processes based on cytoplasmic streaming and to a lesser extent on mass flow (Cooper and Tinker, 1981). The molecular components involved in the eﬄux of P across the arbuscular membrane are not known. The eﬄux of Pi from arbuscules might be connected to the degree of arbuscule formation and/or vacuolar poly Pi hydrolysis in the fungal cells (**Figure 4**). Phosphatases are responsible for the conversion of Pi-esters into Pi in the fungal vacuoles prior to their export. Non-specific alkaline and acid phosphatases (ALPase and ACPase, respectively) have been identified in the vacuole of AMF (Gianinazzi-Pearson and Gianinazzi, 1978; Tisserant et al., 1993; Ezawa et al., 1995, 1999). Most of their activities were insoluble in aequous media, possibly due to the association of the enzymes with the tonoplast, as also shown for yeast (Gianinazzi et al., 1979; Klionsky et al., 1990; Ezawa et al., 1999). Immunolabeling studies indicated that the periarbuscular membrane of the plant contains high H^+^-ATPase activity (Gianinazzi-Pearson et al., 2000). This supports the idea of a plant Pi transporter being active at the periarbuscular membrane surrounding the arbuscule.

Several plant P transporters appear to be responsible for the uptake of P released by mycorrhizal fungi (Daram et al., 1998; Rosewarne et al., 1999; Liu et al., 2002). Characterization of the Pi transporters StPT3 from potato and MtPT4 from *M. truncatula* provides molecular and biochemical evidence for plant Pi uptake at the AM fungus–root interface in AM (Rausch et al., 2001; Harrison et al., 2002). In accordance with Daram et al. (1998) StPT3 functions as a high-affinity Pi transporter, and *StPT3* expression is locally induced upon colonization by the AM fungus *G. intraradices*. The mRNA levels correlated with arbuscule formation in the roots. MtPT4 is exclusively present on the periarbuscular membranes. In contrast, the potato P transporter StPT1 showed high expression in non-mycorrhizal roots, independently of Pi fertilization, while the amount of *StPT2* transcripts was reduced under high-Pi conditions. In mycorrhizal roots, *StPT1* and *StPT2* mRNA concentrations were reduced independently of the Pi treatment. A reduction in Pi transporter mRNA levels upon AMF colonization has also been reported for *M. truncatula MtPT1* and *MtPT2* (Liu et al., 1998b; Chiou et al., 2001; Javot et al., 2007). Therefore, it seems unlikely that these transporters are involved in Pi transfer at the arbuscular interface. The reduced *StPT2* and *MtPT* mRNA levels could either be a local response after colonization, or caused by the increased Pi status of the root or shoot (systemic response; Rausch et al., 2001). The mycorrhiza-responsive plant P transporters are localized on the periarbuscular membrane at the symbiotic interface and not at the arbuscular stalk interface. The localization of these P transporters is regulated by a polarization of the bulk secretary pathway favoring vesicle fusion with the developing periarbuscular membrane rather than with the plasma membrane, which might ensure the proper direction of Pi and other (e.g., sugar) transporters to the peri-arbuscular membrane during arbuscules development (Popova et al., 2010).

Recent advances in genomics and transcriptomics of AMF and other plant beneficial fungi provides a better picture of the fungi-induced biochemical and physiological re-programming of P acquisition mechanisms. *G. intraradices* has a combination of low affinity as well as high affinity Pi transporter genes similar to *Pho91*, and the Na^+^/Pi symporter genes *Pho89* and *GintPT*, respectively, and they are expressed in spores, extra-radical and intra-radical mycelia (Tisserant et al., 2012). The expression pattern of *GintPT* in combination with the arbuscular localization of the GmosPT protein in *G. mosseae* confirmed the involvement, site and role of these P transporters in P delivery to the plant (Benedetto et al., 2005; Balestrini et al., 2007; Gómez-Ariza et al., 2009). The P transporters GintPT, Pho91, and Pho89 are functional at a broad pH range from 4 to 9 and thus active in a variety of acidic and alkaline soils.

**FIGURE 5:**
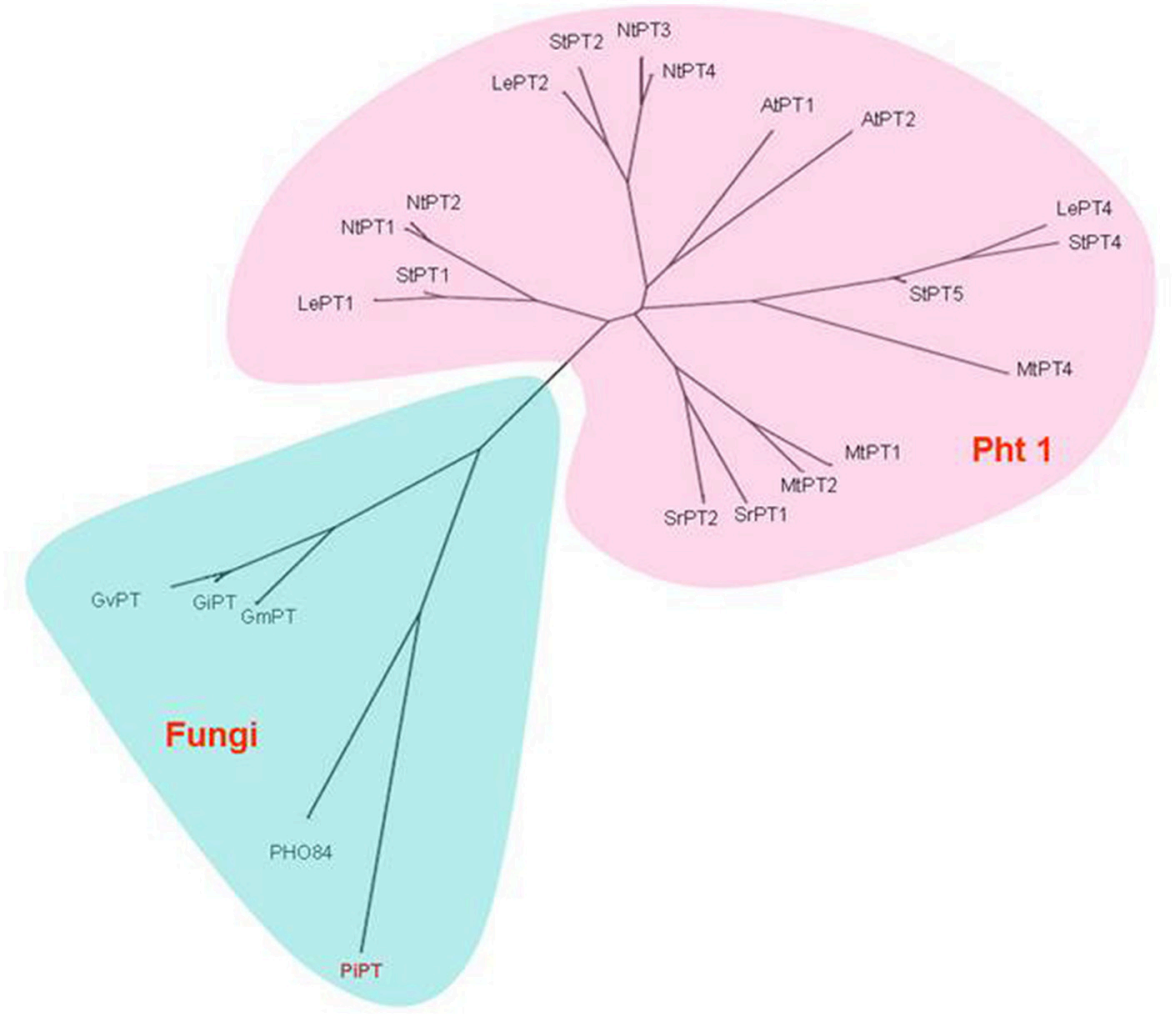
**Unrooted phylogenetic relationship of PiPT to other high affinity Pi transporters from plants and fungi (Yadav et al., 2010)**.

Analysis of the genome sequence of *P. indica* revealed the presence of three putative P transporter genes but only PiPT has been studied yet (Yadav et al., 2010; Zuccaro et al., 2011). PiPT falls in the category of high affinity fungal phosphate transporters and very distinct from phosphate transporters of plants (**Figure 5**). *PiPT* is expressed in extra-radical hyphae under P deprived conditions, similar to transporters from MF (Harrison and van Buuren, 1995; Tatry et al., 2009; Yadav et al., 2010). However, the *P. indica*-mediated mechanism for P delivery is different from that of AMF because *P. indica* does not form arbuscule-like structures (Kumar et al., 2011). Nevertheless, *P. indica* might be an alternative to mycorrhizal fungi and extensive fertilization for crop management strategies. *PiPT* is regulated by the amount of Pi present on the outside of the root, and not by the intracellular Pi pool. The degree of colonization by *P. indica* in maize was similar under low and high Pi concentrations indicating that the colonization is independent of Pi availability (Yadav et al., 2010). Thus, *P. indica* would allow a more efficient Pi uptake independently of the degree of root colonization and other similar responses as in case of AMF colonization (**Table 1**).

**Table 1:** Multiple responses of plants under Pi deficient conditions (Raghothama, 1999).

Kind of Responses	Plant Responses
Morphological responses	Increased root:shoot ratio, changes in root morphology and architecture, increased root hair proliferation, root hair elongation, accumulation of pigments, proteoid roots, increased association with mycorrhizal fungi
Physiological responses	Enhanced Pi uptake, reduced Pi eﬄux, increased Pi use efficiency, mobilization of Pi from the vacuole to cytoplasm, increased translocation of Pi within plants, retention of more Pi in roots, secretion of organic acids, protons and chelators, secretion of phosphatases and RNases, altered respiration, carbon metabolism, photosynthesis, nitrogen fixation, and aromatic enzyme pathways
Biochemical responses	Activation of enzymes, enhanced production of phosphatases, RNases and organic acids, changes in protein phosphorylation, activation of glycolytic bypass pathway
Molecular responses	Activation of genes (RNases, phosphatases, phosphate transporters, Ca-ATPase, vegetative storage proteins, β-glucosidase).

## Pi Mobilization and *P. indica*: Future Prospects for Agricultural Application?

*P. indica* (**Box 2**) produces significant amounts of acid phosphatases for the mobilization of a broad range of insoluble forms of Pi, enabling the host plant to access adequate P from immobilized reserves in the soil (Malla, 2005). The potential of this root-endophytic fungus in improving plant performance and its nutritional status has been demonstrated by Yadav et al. (2010) for maize, by characterizing and analyzing the role of PiPT in *P. indica*-colonized roots. Similar findings were described by Shahollari et al. (2005) who showed that *P. indica* increases the Pi uptake two–threefold in *Arabidopsis* seedlings. Whether this can be generalized, and is valid for all crop species, need to be investigated. Barazani et al. (2005) and Achatz et al. (2010) reported that *P. indica* is not involved in P acquisition and thus increased biomass production in barley and *Nicotiana attenuate*, respectively.

BOX 2 ***Piriformospora indica*: Magic without a Wand.**

An axenically cultivable AMF like-fungus named *P. indica* has been discovered in the Indian Thar desert (Verma et al., 1998). *P. indica* colonizes dicot as well as monocot plants including members of the Brassicaceae, like *Arabidopsis*, which are not colonized by AMF (Shahollari et al., 2005). Colonization of *P. indica* with the medicinal and other economically important plants results in the increased growth yield, high salt tolerance, disease resistance, and nutrition capabilities of the host plant. Because of its beneficial nature this fungus has been termed as plant probiotic (Peškan-Berghöfer et al., 2004; Aschheim et al., 2005; Waller et al., 2005; Kumar et al., 2009).

Efficient transformation systems for *P. indica* has been established by two groups (Yadav et al., 2010; Lahrmann et al., 2013) which allows now functional analyses and the identification of new regulatory genes/proteins controlling Pi acquisition and transfer from the fungus to the host. The availability of the genome sequence of *P. indica* further helps in the identification of novel players in this scenario. As far as we know, *P. indica* can associate with the roots of all plant species tested so far. Furthermore, Sebacinales have been identified around the globe, and *P. indica* transfers benefits to plants under quite different climate, temperature and growth conditions. The fungus can grow axenically and does not need a host for growth and large-scale propagation. An increasing body of studies on this fungus also provides a nice scientific basement for agricultural application. Pi limitation results in major reprogramming of plant developmental processes and they are linked to many signaling pathways influencing plant development in symbiotic interactions. Besides stimulation of biomass, *P. indica* confers tolerance to biotic and abiotic stresses, and can be used as biocontrol agent (Sun et al., 2014). These examples suggest a high potential of the fungus for biotechnological and agricultural applications, in particular under Pi limitation.

## Conflict of Interest Statement

The authors declare that the research was conducted in the absence of any commercial or financial relationships that could be construed as a potential conflict of interest.
